# Supplementary material for: Adverse drug reactions in tuberculosis treatment: Incidence, duration and resolution pathways from a mixed-methods patient-centric study in India
Source: PLOS Glob Public Health. 2025 Dec 29;5(12):e0004149. doi: 10.1371/journal.pgph.0004149 (PMC12747411; doi:10.1371/journal.pgph.0004149)

S1 Appendix: Additional tables and figures

Adverse Drug Reactions in Tuberculosis Treatment: Incidence, Reporting and Outcomes; Insights from a Mixed-Methods Study Across Eight Indian Cities

Section A

Sampling Approach & Size

- ***Purposive Sampling Approach – Qualitative Phase***

Patients: A total of 40 patients were finally interviewed across three districts – Delhi, Jaipur, and Bhopal, with at least 10 patients from each site. Participants were selected basis socio-demographic characteristics (age, gender, stage of TB treatment, profession, and marital status), and stage of treatment (Fig 2). Three stages were recognized, a) the intensive phase or the first two months, b) the continuous phase or when the patients have completed at least 2 months of treatment, and c) post treatment completion. The selection prioritized patients who have faced more challenges during treatment, in order to further insights around resolution management.
Family Members: A total of 5 family members of patients were interviewed across the three sites, and questions elicited information on the type and extent of support provided by family members.
Treatment Coordinators**:** A total of 20 TCs were interviewed (Jaipur- 4, Delhi – 11, Bhopal - 5). The TCs were selected basis their performance – distributed across high performing (10), average/new and low-performing ones (11). The patients who were interviewed also included patients who were being guided by the TCs from these 2 categories. The feedbacks for TCs were drawn from program leaders.
Doctors: A total of 12 doctors were interviewed (Jaipur - 5, Delhi - 4, Bhopal - 3), with questions covering their perception of the JEET PPSA model, and patient management strategies.
Hub Agents: A total of 8 hub agents, who had worked with >=10 patients, were interviewed (Jaipur - 3, Delhi - 4 and Bhopal - 1).
Program Staff: Two program directors and two field staff were interviewed to offer insights around programmatic operations and provider engagement strategies.

- ***Sample Size***

Since our variable of interest is the proportion of population, we used the following formula for estimating required sample size[1].

$$n = \frac{(z)^2\, p(1-p)}{E^2 + \left(z^2 * p * (1-p)/N\right)}$$

whereby,
n = calculated sample size,
N = underlying population
z = confidence level at the chosen probability level (1.96 for 95%),
p = anticipated response distribution (we assume a conservative maximum estimate of 50%), and
E = tolerable margin of error

We have segregated population and sample count as per intensive and continuous phase. We decide on their individual sample sizes by using proportional stratification method, wherein the sample size of each stratum or treatment phase is proportionate to the population size of that treatment phase or stratum. Specifically, we make use of the following equation:

$$n(i) = \frac{N(i) * n}{N}$$

n(i) = sample size of the intensive/continuous phase,
N(i) = patient base of the intensive/continuous phase,
N = total patient base
n = total sample size computed using prescribed C.I. and margin of error

[1]Sampling Design & Analysis, S. Lohr, 1999, Chapter 2, equation 2.17

Section B

Definitions of Treatment Outcomes for drug susceptible TB patients; as per Nikshay[2]

Treatment outcome	Definition	Study outcome	Considered for the study
Cured	Microbiologically confirmed TB patients at the beginning of treatment who was smear or culture negative at the end of the complete treatment	Successful treatment	Yes
Treatment complete	Completed treatment without evidence of failure or clinical deterioration but with no record that the smear or culture results of biological specimen in the last month of treatment was negative		
Lost to follow up	Treatment was interrupted for one consecutive month or more	Unsuccessful treatment	
Died	Died during the course of anti-TB treatment		
Treatment failure	Biological specimen is positive by smear or culture at end of treatment		
Not evaluated	Patients for whom no treatment outcome is assigned; also includes former transfer outs	Other outcomes (not considered)	No
Treatment regimen changed	A TB patient who is on first line regimen and has been diagnosed as having DR TB and switched to drug resistant TB regimen prior to being declared as failed		
Wrongly diagnosed	A patient who is wrongly diagnosed of TB		
Transferred	A patient who has transferred to another facility or state, prior to the outcome being declared		

[2] Central TB Division. Treatment Outcomes for drug susceptible TB patients. In: Technical and Operational Guidelines for TB Control in India. 2016. p. 65.

Section C

Demographic Characteristics of Distinct Patient Profiles; N = 2124 patients

Characteristic	dependents N = 440	elderly dependents N = 216	employed females N = 144	employed males N = 748	homemaker N = 491	other N = 85	p-value
**Males**	230 (52%)	125 (58%)	0 (0%)	748 (100%)	0 (0%)	58 (68%)	<0.001
**Age Category**							<0.001
1. <=22	325 (74%)	0 (0%)	39 (27%)	87 (12%)	64 (13%)	26 (31%)	
2. 23-59	115 (26%)	0 (0%)	100 (69%)	604 (81%)	427 (87%)	59 (69%)	
3. >=60	0 (0%)	216 (100%)	5 (3.5%)	57 (7.6%)	0 (0%)	0 (0%)	
**Age**							<0.001
Median (IQR)	20 (18, 23)	65 (62, 72)	28 (22, 35)	37 (27, 50)	30 (25, 43)	33 (22, 48)	
Mean	23	68	30	39	34	34	
SD	10	7	11	13	11	13	
**Extra Pulmonary**	141 (32%)	69 (32%)	56 (39%)	191 (26%)	182 (37%)	19 (22%)	<0.001
**District**							0.077
ahmedabad	62 (14%)	56 (26%)	21 (15%)	136 (18%)	110 (22%)	18 (21%)	
bhopal	31 (7.0%)	21 (9.7%)	26 (18%)	67 (9.0%)	39 (7.9%)	8 (9.4%)	
darbhanga	50 (11%)	29 (13%)	4 (2.8%)	109 (15%)	56 (11%)	11 (13%)	
delhi	88 (20%)	24 (11%)	28 (19%)	82 (11%)	54 (11%)	7 (8.2%)	
gurgaon	44 (10%)	10 (4.6%)	16 (11%)	73 (9.8%)	39 (7.9%)	5 (5.9%)	
indore	45 (10%)	20 (9.3%)	18 (12%)	83 (11%)	64 (13%)	9 (11%)	
jaipur	64 (15%)	39 (18%)	10 (6.9%)	87 (12%)	77 (16%)	16 (19%)	
surat	56 (13%)	17 (7.9%)	21 (15%)	111 (15%)	52 (11%)	11 (13%)	

Note: p values are given for Kruskal-Wallis rank sum test

Section D

Age related risk factors for individual ADRs

Table D.1

Tukey Tests; Significance of difference in means for incidence of distinct ADRs reported among distinct age groups; N = 2124 patients

	**Age Group Pair**	*Adjusted P (Sidak correction)*
**diarrhoea**	23-59 &<=22 years	0.894
	>=60 &<=22 years	0.0002*
	>=60 & 23-59 years	0.0001*
**drug fever**	23-59 &<=22 years	0.069
	>=60 &<=22 years	0.053
	>=60 & 23-59 years	0.650
**itching**	23-59 &<=22 years	0.085
	>=60 &<=22 years	0.277
	>=60 & 23-59 years	0.998
**joint pains**	23-59 &<=22 years	0.0011*
	>=60 &<=22 years	0.0143*
	>=60 & 23-59 years	0.928
**trepidation**	23-59 &<=22 years	0.738
	>=60 &<=22 years	0.542
	>=60 & 23-59 years	0.818
**vomit**	23-59 &<=22 years	0.0003*
	>=60 &<=22 years	0.068
	>=60 & 23-59 years	0.845
**acne**	23-59 &<=22 years	0.000*
	>=60 &<=22 years	0.000*
	>=60 & 23-59 years	0.184
**fatigue**	23-59 &<=22 years	0.577
	>=60 &<=22 years	0.226
	>=60 & 23-59 years	0.535
**hair loss**	23-59 &<=22 years	0.000*
	>=60 &<=22 years	0.000*
	>=60 & 23-59 years	0.0365*
**skin darkening**	23-59 &<=22 years	0.0067*
	>=60 &<=22 years	0.007*
	>=60 & 23-59 years	0.557
**teeth darkening**	23-59 &<=22 years	0.957
	>=60 &<=22 years	0.0437*
	>=60 & 23-59 years	0.0372*

Note: a) p value gives the results from Tukey test to test the significance of difference in ADR incidence for distinct age groups; b) * or a significant value shows that the paired age-groups are statistically significantly different from each other with a p value of lower than 0.05

Section E

Duration of ADRs

Table E.1

Summary of duration of active ADRs; N = 1729 (those who experienced at least one active ADR)

	Age Class				Gender			Categories							Site of Disease		
	**<=22**	**23-59**	**>=60**	*p-value*	**female**	**male**	*p-value*	**dependents**	**elderly dependents**	**employed females**	**employed males**	**homemaker**	**other**	*p-value*	**pulmonary**	**extra pulmonary**	*p-value*
**#Patients**	444	1063	222		822	907		347	174	126	584	426	72		1198	531	
**diarrhoea**				0.9			0.3							0.7			<0.001
<=1 week	17 (45%)	53 (53%)	23 (55%)		38 (48%)	55 (54%)		17 (55%)	14 (47%)	7 (64%)	34 (52%)	16 (44%)	5 (71%)		83 (61%)	10 (22%)	
2-3 weeks	11 (29%)	20 (20%)	7 (17%)		15 (19%)	23 (23%)		8 (26%)	5 (17%)	0 (0%)	15 (23%)	9 (25%)	1 (14%)		25 (19%)	13 (29%)	
1 month	9 (24%)	23 (23%)	9 (21%)		23 (29%)	18 (18%)		6 (19%)	8 (27%)	4 (36%)	14 (22%)	9 (25%)	0 (0%)		22 (16%)	19 (42%)	
1.5 months	0 (0%)	3 (3.0%)	2 (4.8%)		2 (2.5%)	3 (3.0%)		0 (0%)	2 (6.7%)	0 (0%)	1 (1.5%)	2 (5.6%)	0 (0%)		3 (2.2%)	2 (4.4%)	
2 months	0 (0%)	1 (1.0%)	0 (0%)		0 (0%)	1 (1.0%)		0 (0%)	0 (0%)	0 (0%)	0 (0%)	0 (0%)	1 (14%)		1 (0.7%)	0 (0%)	
>=2 months	1 (2.6%)	0 (0%)	1 (2.4%)		1 (1.3%)	1 (1.0%)		0 (0%)	1 (3.3%)	0 (0%)	1 (1.5%)	0 (0%)	0 (0%)		1 (0.7%)	1 (2.2%)	
**drug fever**				0.5			0.13							0.5			0.15
<=1 week	74 (31%)	173 (34%)	37 (37%)		129 (32%)	155 (36%)		57 (32%)	27 (36%)	25 (34%)	97 (35%)	61 (31%)	17 (46%)		208 (34%)	76 (33%)	
2-3 weeks	58 (24%)	131 (26%)	24 (24%)		96 (24%)	117 (27%)		49 (28%)	18 (24%)	14 (19%)	82 (30%)	45 (23%)	5 (14%)		161 (26%)	52 (23%)	
1 month	54 (23%)	88 (18%)	19 (19%)		91 (22%)	70 (16%)		33 (19%)	17 (22%)	16 (22%)	41 (15%)	46 (23%)	8 (22%)		121 (20%)	40 (17%)	
1.5 months	13 (5.5%)	17 (3.4%)	3 (3.0%)		18 (4.4%)	15 (3.5%)		11 (6.2%)	2 (2.6%)	3 (4.1%)	8 (2.9%)	8 (4.0%)	1 (2.7%)		25 (4.1%)	8 (3.5%)	
2 months	9 (3.8%)	37 (7.4%)	5 (5.1%)		27 (6.7%)	24 (5.5%)		2 (1.1%)	4 (5.3%)	8 (11%)	15 (5.4%)	18 (9.0%)	4 (11%)		32 (5.3%)	19 (8.3%)	
>=2 months	30 (13%)	56 (11%)	11 (11%)		45 (11%)	52 (12%)		25 (14%)	8 (11%)	8 (11%)	33 (12%)	21 (11%)	2 (5.4%)		62 (10%)	35 (15%)	
**itching**				0.8			0.6							0.2			0.8
<=1 week	69 (44%)	126 (40%)	29 (43%)		108 (39%)	116 (44%)		46 (41%)	20 (38%)	25 (57%)	75 (43%)	52 (37%)	6 (38%)		148 (42%)	76 (41%)	
2-3 weeks	32 (20%)	88 (28%)	20 (30%)		74 (27%)	66 (25%)		25 (22%)	16 (31%)	10 (23%)	44 (25%)	42 (30%)	3 (19%)		92 (26%)	48 (26%)	
1 month	33 (21%)	58 (18%)	8 (12%)		58 (21%)	41 (15%)		23 (20%)	7 (13%)	7 (16%)	29 (17%)	30 (21%)	3 (19%)		68 (19%)	31 (17%)	
1.5 months	10 (6.4%)	10 (3.2%)	3 (4.5%)		14 (5.1%)	9 (3.4%)		9 (8.0%)	3 (5.8%)	0 (0%)	5 (2.9%)	5 (3.5%)	1 (6.2%)		15 (4.2%)	8 (4.3%)	
2 months	4 (2.5%)	12 (3.8%)	3 (4.5%)		10 (3.6%)	9 (3.4%)		1 (0.9%)	3 (5.8%)	1 (2.3%)	6 (3.4%)	7 (4.9%)	1 (6.2%)		11 (3.1%)	8 (4.3%)	
>=2 months	9 (5.7%)	23 (7.3%)	4 (6.0%)		11 (4.0%)	25 (9.4%)		9 (8.0%)	3 (5.8%)	1 (2.3%)	15 (8.6%)	6 (4.2%)	2 (12%)		22 (6.2%)	14 (7.6%)	
**joint pain**				>0.9			0.092							0.2			0.4
<=1 week	59 (28%)	198 (31%)	43 (31%)		135 (28%)	165 (33%)		50 (31%)	33 (31%)	17 (24%)	114 (33%)	70 (27%)	16 (40%)		203 (30%)	97 (32%)	
2-3 weeks	61 (29%)	150 (24%)	28 (20%)		111 (23%)	128 (25%)		40 (25%)	24 (22%)	24 (33%)	86 (25%)	55 (21%)	10 (25%)		166 (25%)	73 (24%)	
1 month	50 (23%)	143 (23%)	36 (26%)		125 (26%)	104 (21%)		41 (25%)	26 (24%)	16 (22%)	72 (21%)	71 (27%)	3 (7.5%)		149 (22%)	80 (26%)	
1.5 months	16 (7.5%)	26 (4.1%)	5 (3.6%)		21 (4.4%)	26 (5.1%)		8 (5.0%)	4 (3.7%)	2 (2.8%)	16 (4.7%)	13 (5.0%)	4 (10%)		37 (5.5%)	10 (3.3%)	
2 months	14 (6.6%)	45 (7.1%)	10 (7.2%)		38 (7.9%)	31 (6.1%)		9 (5.6%)	6 (5.6%)	4 (5.6%)	21 (6.2%)	25 (9.6%)	4 (10%)		50 (7.4%)	19 (6.2%)	
>=2 months	13 (6.1%)	70 (11%)	16 (12%)		48 (10%)	51 (10%)		13 (8.1%)	15 (14%)	9 (12%)	32 (9.4%)	27 (10%)	3 (7.5%)		71 (11%)	28 (9.1%)	
**trepidation**				0.2			0.6							0.6			0.5
<=1 week	122 (46%)	325 (49%)	62 (42%)		270 (48%)	239 (47%)		89 (45%)	49 (43%)	42 (52%)	160 (48%)	141 (46%)	28 (60%)		350 (47%)	159 (48%)	
2-3 weeks	63 (24%)	172 (26%)	43 (29%)		148 (26%)	130 (25%)		54 (27%)	30 (26%)	18 (22%)	87 (26%)	86 (28%)	3 (6.4%)		187 (25%)	91 (27%)	
1 month	48 (18%)	101 (15%)	19 (13%)		88 (16%)	80 (16%)		35 (18%)	16 (14%)	11 (14%)	48 (15%)	49 (16%)	9 (19%)		118 (16%)	50 (15%)	
1.5 months	13 (4.9%)	26 (3.9%)	9 (6.1%)		19 (3.4%)	29 (5.7%)		9 (4.5%)	8 (7.0%)	0 (0%)	19 (5.8%)	9 (3.0%)	3 (6.4%)		39 (5.2%)	9 (2.7%)	
2 months	10 (3.8%)	18 (2.7%)	4 (2.7%)		15 (2.7%)	17 (3.3%)		5 (2.5%)	4 (3.5%)	5 (6.2%)	8 (2.4%)	7 (2.3%)	3 (6.4%)		21 (2.8%)	11 (3.3%)	
>=2 months	9 (3.4%)	22 (3.3%)	10 (6.8%)		25 (4.4%)	16 (3.1%)		7 (3.5%)	8 (7.0%)	4 (5.0%)	8 (2.4%)	13 (4.3%)	1 (2.1%)		29 (3.9%)	12 (3.6%)	
**vomit**				0.3			0.067							0.2			0.6
<=1 week	143 (64%)	272 (66%)	54 (58%)		242 (61%)	227 (68%)		112 (63%)	43 (57%)	36 (62%)	133 (71%)	124 (62%)	21 (68%)		342 (65%)	127 (63%)	
2-3 weeks	52 (23%)	87 (21%)	23 (25%)		98 (25%)	64 (19%)		42 (24%)	16 (21%)	11 (19%)	34 (18%)	51 (25%)	8 (26%)		114 (22%)	48 (24%)	

1 month	20 (9.0%)	34 (8.2%)	6 (6.5%)		36 (9.1%)	24 (7.2%)		18 (10%)	6 (8.0%)	7 (12%)	12 (6.4%)	16 (8.0%)	1 (3.2%)		43 (8.2%)	17 (8.4%)	
1.5 months	3 (1.3%)	6 (1.4%)	4 (4.3%)		8 (2.0%)	5 (1.5%)		3 (1.7%)	4 (5.3%)	0 (0%)	2 (1.1%)	3 (1.5%)	1 (3.2%)		8 (1.5%)	5 (2.5%)	
2 months	2 (0.9%)	8 (1.9%)	3 (3.2%)		8 (2.0%)	5 (1.5%)		1 (0.6%)	3 (4.0%)	0 (0%)	3 (1.6%)	6 (3.0%)	0 (0%)		10 (1.9%)	3 (1.5%)	
>=2 months	3 (1.3%)	7 (1.7%)	3 (3.2%)		5 (1.3%)	8 (2.4%)		1 (0.6%)	3 (4.0%)	4 (6.9%)	4 (2.1%)	1 (0.5%)	0 (0%)		10 (1.9%)	3 (1.5%)	

Note: p values are given for Kruskal-Wallis rank sum test; N (%) tells the number (share) of patients who experienced the ADR for the said duration

Table E.2

Summary of duration of passive ADRs; N = 1333 (those who experienced at least one passive ADR)

	Age Class				Gender			Categories							Site of Disease		
	**<=22**	**23-59**	**>=60**	*p-value*	**female**	**male**	*p-value*	**dependents**	**elderly dependents**	**employed females**	**employed males**	**homemaker**	**other**	*p-value*	**pulmonary**	**extra pulmonary**	*p-value*
**#Patients N>**	**373**	**807**	**153**		**660**	**673**		**291**	**124**	**101**	**417**	**341**	**59**		**925**	**408**	
**fatigue**				0.2			0.2							0.2			0.5
<=2 weeks	98 (32%)	210 (29%)	38 (27%)		188 (33%)	158 (26%)		71 (30%)	32 (28%)	23 (26%)	98 (26%)	101 (34%)	21 (38%)		249 (30%)	97 (28%)	
1 month	98 (32%)	202 (28%)	40 (28%)		151 (26%)	189 (31%)		79 (33%)	33 (28%)	22 (25%)	118 (32%)	74 (25%)	14 (25%)		239 (29%)	101 (29%)	
1.5 months	61 (20%)	164 (23%)	40 (28%)		118 (21%)	147 (24%)		50 (21%)	32 (28%)	18 (20%)	88 (24%)	63 (21%)	14 (25%)		183 (22%)	82 (24%)	
2 months	23 (7.4%)	43 (6.0%)	4 (2.8%)		35 (6.1%)	35 (5.8%)		15 (6.3%)	2 (1.7%)	9 (10%)	23 (6.1%)	17 (5.7%)	4 (7.1%)		52 (6.3%)	18 (5.2%)	
2.5 months	9 (2.9%)	17 (2.4%)	8 (5.6%)		18 (3.2%)	16 (2.7%)		6 (2.5%)	5 (4.3%)	2 (2.2%)	9 (2.4%)	10 (3.3%)	2 (3.6%)		24 (2.9%)	10 (2.9%)	
>=3 months	22 (7.1%)	81 (11%)	13 (9.1%)		60 (11%)	56 (9.3%)		16 (6.8%)	12 (10%)	15 (17%)	38 (10%)	34 (11%)	1 (1.8%)		78 (9.5%)	38 (11%)	
**hair loss**				0.004			<0.001							0.08			0.054
<=2 weeks	25 (17%)	64 (27%)	12 (38%)		58 (20%)	43 (34%)		19 (16%)	8 (33%)	10 (23%)	25 (34%)	35 (24%)	4 (33%)		71 (25%)	30 (22%)	
1 month	33 (22%)	57 (24%)	8 (25%)		67 (23%)	31 (25%)		28 (24%)	6 (25%)	8 (18%)	18 (24%)	37 (26%)	1 (8.3%)		72 (26%)	26 (19%)	
1.5 months	52 (34%)	64 (27%)	9 (28%)		94 (32%)	31 (25%)		42 (36%)	7 (29%)	11 (25%)	18 (24%)	41 (28%)	6 (50%)		85 (30%)	40 (30%)	
2 months	15 (9.9%)	19 (8.1%)	0 (0%)		28 (9.6%)	6 (4.8%)		8 (6.8%)	0 (0%)	7 (16%)	4 (5.4%)	14 (9.7%)	1 (8.3%)		19 (6.7%)	15 (11%)	
2.5 months	9 (6.0%)	7 (3.0%)	0 (0%)		12 (4.1%)	4 (3.2%)		10 (8.5%)	0 (0%)	1 (2.3%)	0 (0%)	5 (3.4%)	0 (0%)		10 (3.5%)	6 (4.4%)	
>=3 months	17 (11%)	23 (9.8%)	3 (9.4%)		33 (11%)	10 (8.0%)		11 (9.3%)	3 (12%)	7 (16%)	9 (12%)	13 (9.0%)	0 (0%)		25 (8.9%)	18 (13%)	
**skin darkening**				0.3			0.1							0.6			0.2
<=2 weeks	34 (30%)	56 (28%)	14 (39%)		49 (27%)	55 (33%)		23 (28%)	10 (40%)	8 (29%)	38 (33%)	25 (27%)	0 (0%)		71 (30%)	33 (28%)	
1 month	30 (26%)	60 (30%)	10 (28%)		50 (27%)	50 (30%)		25 (30%)	7 (28%)	5 (18%)	31 (27%)	26 (29%)	6 (60%)		71 (30%)	29 (25%)	
1.5 months	23 (20%)	43 (21%)	8 (22%)		40 (22%)	34 (20%)		19 (23%)	6 (24%)	5 (18%)	21 (18%)	19 (21%)	4 (40%)		50 (21%)	24 (21%)	
2 months	14 (12%)	17 (8.4%)	1 (2.8%)		22 (12%)	10 (5.9%)		9 (11%)	0 (0%)	4 (14%)	8 (6.9%)	11 (12%)	0 (0%)		18 (7.6%)	14 (12%)	
2.5 months	2 (1.7%)	9 (4.5%)	0 (0%)		5 (2.7%)	6 (3.6%)		1 (1.2%)	0 (0%)	2 (7.1%)	6 (5.2%)	2 (2.2%)	0 (0%)		6 (2.5%)	5 (4.3%)	
>=3 months	12 (10%)	17 (8.4%)	3 (8.3%)		18 (9.8%)	14 (8.3%)		6 (7.2%)	2 (8.0%)	4 (14%)	12 (10%)	8 (8.8%)	0 (0%)		20 (8.5%)	12 (10%)	
**acne**				0.5			0.8							0.035			0.5
<=2 weeks	27 (36%)	37 (37%)	5 (42%)		43 (40%)	26 (32%)		16 (27%)	3 (38%)	9 (39%)	14 (30%)	22 (52%)	5 (62%)		42 (33%)	27 (44%)	
1 month	21 (28%)	31 (31%)	5 (42%)		28 (26%)	29 (36%)		17 (28%)	5 (62%)	6 (26%)	17 (37%)	10 (24%)	2 (25%)		42 (33%)	15 (25%)	
1.5 months	15 (20%)	22 (22%)	2 (17%)		21 (20%)	18 (22%)		14 (23%)	0 (0%)	6 (26%)	12 (26%)	6 (14%)	1 (12%)		30 (24%)	9 (15%)	
2 months	7 (9.2%)	2 (2.0%)	0 (0%)		7 (6.5%)	2 (2.5%)		8 (13%)	0 (0%)	0 (0%)	1 (2.2%)	0 (0%)	0 (0%)		6 (4.8%)	3 (4.9%)	
2.5 months	4 (5.3%)	3 (3.0%)	0 (0%)		4 (3.7%)	3 (3.8%)		3 (5.0%)	0 (0%)	1 (4.3%)	1 (2.2%)	2 (4.8%)	0 (0%)		3 (2.4%)	4 (6.6%)	
>=3 months	2 (2.6%)	4 (4.0%)	0 (0%)		4 (3.7%)	2 (2.5%)		2 (3.3%)	0 (0%)	1 (4.3%)	1 (2.2%)	2 (4.8%)	0 (0%)		3 (2.4%)	3 (4.9%)	
**teeth**				>0.9			>0.9							0.051			0.5
<=2 weeks	15 (54%)	43 (60%)	15 (58%)		34 (58%)	39 (58%)		13 (52%)	11 (65%)	2 (25%)	24 (55%)	20 (69%)	3 (100%)		52 (57%)	21 (62%)	

1 month	9 (32%)	13 (18%)	8 (31%)	15 (25%)	15 (22%)	6 (24%)	4 (24%)	2 (25%)	11 (25%)	7 (24%)	0 (0%)	21 (23%)	9 (26%)
1.5 months	3 (11%)	14 (19%)	3 (12%)	8 (14%)	12 (18%)	6 (24%)	2 (12%)	2 (25%)	8 (18%)	2 (6.9%)	0 (0%)	17 (18%)	3 (8.8%)
2 months	0 (0%)	2 (2.8%)	0 (0%)	1 (1.7%)	1 (1.5%)	0 (0%)	0 (0%)	1 (12%)	1 (2.3%)	0 (0%)	0 (0%)	1 (1.1%)	1 (2.9%)
>=3 months	1 (3.6%)	0 (0%)	0 (0%)	1 (1.7%)	0 (0%)	0 (0%)	0 (0%)	1 (12%)	0 (0%)	0 (0%)	0 (0%)	1 (1.1%)	0 (0%)

Note: a) p values are given for Kruskal-Wallis rank sum test, b) N (%) tells the number (share) of patients who experienced the ADR for the said duration

Section F

ADR Management; Resolutions taken by distinct patient types

Table F.1

Summary for Active ADR management by distinct patient types

	Age Class				Gender			Categories							Site of Disease		
	**<=22**	**23-59**	**>=60**	*p-value*	**female**	**male**	*p-value*	**dependents**	**elderly dependents**	**employed females**	**employed males**	**homemaker**	**other**	*p-value*	**pulmonary**	**extra pulmonary**	*p-value*
**#Patients N>**	444	1063	222		822	907		347	174	126	584	426	72		1198	531	
called doctor	121 (27%)	305 (29%)	70 (32%)	0.5	255 (31%)	241 (27%)	0.041	110 (32%)	56 (32%)	39 (31%)	152 (26%)	124 (29%)	15 (21%)	0.2	334 (28%)	162 (31%)	0.3
went to the doctor	216 (49%)	519 (49%)	120 (54%)	0.3	417 (51%)	438 (48%)	0.3	165 (48%)	90 (52%)	66 (52%)	288 (49%)	207 (49%)	39 (54%)	0.8	604 (50%)	251 (47%)	0.2
home remedy	48 (11%)	104 (9.8%)	24 (11%)	0.8	70 (8.5%)	106 (12%)	0.029	37 (11%)	16 (9.2%)	12 (9.5%)	65 (11%)	41 (9.6%)	5 (6.9%)	0.9	127 (11%)	49 (9.2%)	0.4
asked TC	188 (42%)	476 (45%)	68 (31%)	<0.001	372 (45%)	360 (40%)	0.019	143 (41%)	53 (30%)	60 (48%)	242 (41%)	204 (48%)	30 (42%)	0.004	499 (42%)	233 (44%)	0.4
asked compounder	0 (0%)	1 (<0.1%)	0 (0%)	0.7	1 (0.1%)	0 (0%)	0.3	0 (0%)	0 (0%)	0 (0%)	0 (0%)	1 (0.2%)	0 (0%)	0.7	1 (<0.1%)	0 (0%)	0.5
did nothing	89 (20%)	225 (21%)	46 (21%)	0.9	151 (18%)	209 (23%)	0.017	65 (19%)	39 (22%)	22 (17%)	136 (23%)	81 (19%)	17 (24%)	0.4	246 (21%)	114 (21%)	0.7

Note: *a) Patient Ns in top row represent the number of patients who experienced active ADRs, b) p values are given for Kruskal-Wallis test, testing significance in differences of management adopted by patients, c) N (%) tells the number (share) of patients who adopted the said management option*

Table F.2: Summary for Passive ADR management by distinct patient types

	Age Class				Gender			Categories							Site of Disease		
	**<=22**	**23-59**	**>=60**	*p-value*	**female**	**male**	*p-value*	**dependents**	**elderly dependents**	**employed females**	**employed males**	**homemaker**	**other**	*p-value*	**pulmonary**	**extra pulmonary**	*p-value*
**#Patients N>**	373	807	153		660	673		291	124	101	417	341	59		925	408	
asked doctor	190 (51%)	419 (52%)	85 (56%)	0.6	347 (53%)	347 (52%)	0.7	156 (54%)	63 (51%)	54 (53%)	217 (52%)	172 (50%)	32 (54%)	>0.9	488 (53%)	206 (50%)	0.4
consulted another doctor	55 (15%)	93 (12%)	25 (16%)	0.13	89 (13%)	84 (12%)	0.6	49 (17%)	20 (16%)	13 (13%)	46 (11%)	40 (12%)	5 (8.5%)	0.2	128 (14%)	45 (11%)	0.2
home remedy	48 (13%)	92 (11%)	24 (16%)	0.3	75 (11%)	89 (13%)	0.3	37 (13%)	16 (13%)	14 (14%)	55 (13%)	37 (11%)	5 (8.5%)	0.8	124 (13%)	40 (9.8%)	0.065
asked TC	155 (42%)	353 (44%)	49 (32%)	0.026	292 (44%)	265 (39%)	0.072	109 (37%)	38 (31%)	49 (49%)	183 (44%)	155 (45%)	23 (39%)	0.021	372 (40%)	185 (45%)	0.08
sked compounder	0 (0%)	1 (0.1%)	0 (0%)	0.7	0 (0%)	1 (0.1%)	0.3	0 (0%)	0 (0%)	0 (0%)	1 (0.2%)	0 (0%)	0 (0%)	0.8	1 (0.1%)	0 (0%)	0.5
did nothing	100 (27%)	226 (28%)	44 (29%)	0.9	178 (27%)	192 (29%)	0.5	71 (24%)	39 (31%)	25 (25%)	121 (29%)	97 (28%)	17 (29%)	0.6	240 (26%)	130 (32%)	0.026
alternative medicine	8 (2.1%)	10 (1.2%)	3 (2.0%)	0.5	8 (1.2%)	13 (1.9%)	0.3	4 (1.4%)	1 (0.8%)	0 (0%)	8 (1.9%)	7 (2.1%)	1 (1.7%)	0.7	17 (1.8%)	4 (1.0%)	0.2

Note: *a) Patient Ns in top row represent the number of patients who experienced passive ADRs, b) p values are given for Kruskal-Wallis test, testing significance in differences of management adopted by patients, c) N (%) tells the number (share) of patients who adopted the said management option*

Table F.3

Passive ADR management; relative to Active ADR management

		Passive ADR Management						
Active ADR Management	N	asked doctor	consulted another doctor	home remedy	asked TC	asked compounder	did nothing	alternative medicine
called doctor	348	74%	13%	11%	53%	0%	14%	2%
went to the doctor	657	67%	19%	12%	40%		15%	2%
home remedy	149	44%	24%	70%	45%		14%	3%
asked TC	551	57%	14%	14%	77%		16%	2%
asked compounder	1			100%				
did nothing	221	13%	5%	9%	20%		82%	

Note: a) N refers to the number of patients who faced an active as well as a passive ADR and reported a corresponding action for both

Section G

Incidence of ADRs among distinct districts

Table G.1

ADR incidence among distinct districts

	**ahmedabad**	**bhopal**	**darbhanga**	**delhi**	**gurgaon**	**indore**	**jaipur N**	**surat**	**p-value**
**N >**	**403**	**192**	**259**	**283**	**187**	**239**	**293**	**268**	
**Total ADRs**	3.01 (2.04)	3.76 (3.04)	3.15 (2.76)	3.96 (2.55)	3.10 (2.31)	2.63 (2.03)	2.96 (2.09)	2.46 (1.99)	<0.001
**Active ADRs**	2.04 (1.39)	2.40 (1.82)	2.00 (1.75)	2.47 (1.69)	2.19 (1.56)	1.73 (1.39)	2.00 (1.44)	1.63 (1.35)	<0.001
**Passive ADRs**	0.97 (1.05)	1.36 (1.46)	1.15 (1.23)	1.49 (1.26)	0.90 (1.09)	0.90 (0.93)	0.96 (0.98)	0.82 (1.03)	<0.001

Note: a) Mean (SD) values are reported, p values for Kruskal-Wallis rank sum test

Figure G.1

Box Plots for ADRs experienced by patients in distinct districts; N = 2124

Notes: 1) Black dots represent raw data. Red dots and error bars represent (estimated marginal) means ± 95% confidence interval per group. Means not sharing any letter are significantly different by the Sidak-test at the 5% level of significance

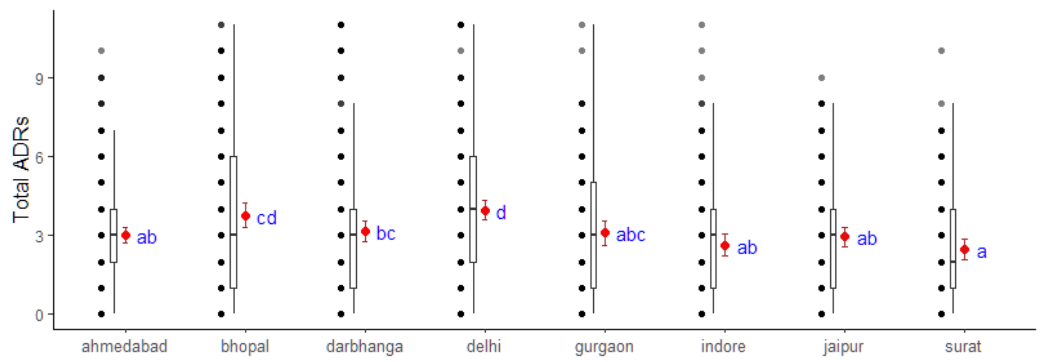

Supplement: S1 Appendix — (PDF) [file pgph.0004149.s001.pdf]
