# Supplementary material for: Adverse drug reactions in tuberculosis treatment: Incidence, duration and resolution pathways from a mixed-methods patient-centric study in India
Source: PLOS Glob Public Health. 2025 Dec 29;5(12):e0004149. doi: 10.1371/journal.pgph.0004149 (PMC12747411; doi:10.1371/journal.pgph.0004149)
Supplement: S2 Appendix — (PDF) [file pgph.0004149.s002.pdf]

FORM- Patient and TC

Please thoroughly go through instructions given against different questions in this document

Responses from both patient and TC

* Required

SECTION 1 (to be filled by TC; TC द्वारा भरने के लिए)

1.1 Name of the city *

Choose

1.2 Name of the TC *

Your answer

1.3 Nikshay ID of patient *

Your answer

A Nikshay ID is usually 8-character. Needs to be filled properly for identification later on (we are not filling names of patients)

1.5 Patients's age (in years) *

Your answer

Age here should be 16 and above since we are only surveying patients within this criteria.
Please do not add "year of birth" instead.

1.6 क्या आपको अकसर पेशेंट की जगह उनके घर में किसी और से बात करनी पड़ती है जैसे की पतिĖ बच्चेĖ माताåपिता या अभिभावक(guardian). *

This is asking that "in general", at other/usual times, who does the TC speak to regarding treatment— patient or someone else.

- Yes
- No

1.7 आगे के प्रश्न किससे पूछे जा रहे हैं? *

This is asking that "currently", at the time of taking survey, who is the TC speaking to

- पेशेंट
- पति׀ पत्नी
- माता׀ पिता
- बेटा׀ बेटी
- Other:

SECTION 2 : काम और जीवन से संबंधित प्रश्न।

पेशेंट (मरीज़) से पूछिए यदि परिवार में किसी और से बात कर रहे है तो सवाल पेशेंट के लिए पूछिए -

2.1 आगे आपके डॉक्टर ने क्या बोला हैå कितने महीने और दवाई खानी पड़ेगी?

Your answer

Here, we want to know what doctor has told the patient regarding "treatment left" from the current time.

2.2 आपकी। इनकी वेवाहिक स्तिथि क्या है? में ऑप्शन दे रहा। रही हूंं इनमे से बता दीजिए *

- कुंवारा I कुँवारी
- शादीशुदा
- तलाक़ या अलग रह रहे है
- विधवा। विदुर

This question is for the Patient.

If TC is speaking to the patient's relative, please ensure that they mark patient's marital status.

2.3 आपके I इनके लिए क्या क्या लागू होता है. मैं optUons बता रहा। रही हूंं इनमें से बता दीजिए. *

- नौकरी
- खुद का काम
- नौकरी भी और ख़ुद का काम भी
- काम ढूंढ रहे हैं
- पढ़ाई करते है (विद्यार्थीIstudent)
- गृहणी (housewife)
- रिटायर्ड (retired)
- Others

This question is ALSO for the Patient.

If TC is speaking to the patient's relative, please ensure that they mark patient's marital status.

SECTION 2 : काम और जीवन से संबंधित प्रश्न।

2.4 कृपया मुझे बताएं की इनमें से आपाइन पर क्या लागू होता है I में options बोल रहाI रही हूं I

- काम ठीक चल रहा है।
- आप बीमारी के कारण छुट्टी पर है, पर सैलरी मिल रही है ।
- आप बीमारी के कारण छुट्टी पर है, और सैलरी नहीं मिल रही या कम हो गयी है ।
- बीमारी की वजह से, नौकरी छूट गई है या काम बंद हो गया है
- COVID की वजह से, नौकरी छूट गई है या काम बंद हो गया है
- बीमारी और COVID, दोनों की ही की वजह से, नौकरी छूट गई है या काम बंद हो गया है
- None of the above

This question is ALSO for the Patient.

If TC is speaking to the patient's relative, please ensure that they mark patient's marital status.

SECTION 3: परिवार के बारे में प्रश्न

(TC पढ़ के सुनाइए) अब, मैं आपसे आपके परिवार के बारे में कुछ प्रश्न करूँगाIकरुँगी I

3.1 आपके परिवार मेंई आपके साथई कितने बड़े लोग रहते है? जो 16 साल या उससे बड़े है? अपने आप को भी मिलाना अगर आप की उम्र 16 साल से बड़ी है (रेकर्ड करे) *

We want to know "how many adults", in total, are there in the house, including the patient.

(Patient will always be 16 or above for this survey because of our selection criteria)

Your answer

3.2 बच्चे कितने हैं? 15 साल या उससे छोटे? (रेकर्ड करे) *

Your answer

3.3 घर में सबसे बड़े सदस्य की उमर कितनी होगी? (TC - पेशंट के जवाब अनुसार ऑप्शन सलेक्ट करें) *

○ 65 साल से कम ; <65

○ 65 साल से ज़्यादा ; >=65

3.4 घर में सबसे छोटे सदस्य की उमर कितनी होगी? (TC - पेशंट के जवाब अनुसार ऑप्शन सलेक्ट करें) *

○ 6 साल से कम , upto 6

○ 7-10 साल के बीच

○ 11 साल या उससे ज़्यादा

SECTION 4 : ट्रीटमेंट की शुरुआत

(TC पढ़ के सुनाइए) अब हम आपसे जानने की कोशिश करेंगे कि जब आपका । इनका इलाज शुरू हुआ, तब आपका । इनका अनुभव कैसा रहा और क्या चिन्ताएँ थी? कृपया निस्संकोच होकर जवाब दें ।

4.1 जब आपको अपनी । इनकी बीमारी के बारे में पता चलाÉ तब आपने निजी या परिवार वालों को बताने के सम्बंध में क्या किया? मैं ऑप्शन दे रहा। रही हूँÉ बताइए जो आपके लिए सही है *

○ आपको काफी संकोच था, किसी को नहीं बताया

○ आपको शुरु में संकोच था पर फिर कुछ लोगों को बताया

○ आपको कोई संकोच नहीं था बताने में

SECTION 4 : ट्रीटमेंट की शुरुआत

4.2 इस बीमारी के बारे में आपने किस को बताया åकरीबी लोग या कोई और? मैं ऑप्शन पढ़ के सुनाती।
सुनाता हूँÉजो जो सही हैÉबताना हाँ या नहींï आप एक से ज्यादा भी हाँ बोल सकते है *

- [ ] घर में सबको
- [ ] कुछ चुने हुए परिवार वालों को I रिश्तेदारों को I जानकार लोगों को
- [ ] जो लोग जानकारी दे सके इलाज के बारे में, उनको बताया
- [ ] जो लोग पैसे के मामले में मदद कर सके, उनको बताया

Here, a minimum of 1 and a maximum of 3 options can be selected.

TC needs to read all the options so patient can select most appropriate

4.3 जब बीमारी के पता लगने के बाद आप घर गए तो लोगों ने बीमारी के बारे में क्या क्या बताया? मैं ऑप्शन दे रही।रहा हूँÉइनमें हर एक के लिए बताना की आपको किसी ने ये बताया की नहीं। आप एक से ज्यादा भी हाँ बोल सकते है *

- [ ] रिश्तेदारी में किसी को हुई थी
- [ ] बर्तन, खाना अलग करना पड़ेगा
- [ ] आजकल तो किसी को भी हो जाती है, दवाई से ठीक हो जाएगी
- [ ] छूत की बीमारी है
- [ ] जानलेवा बीमारी है
- [ ] देसी इलाज ज़्यादा फ़ायदा करेगा
- [ ] Other:

Here, any number of options can be selected

TC needs to read all the options so patient can select most appropriate

A SQUARE means we can choose more than one option for the question

SECTION 5 : ट्रीटमेंट के कारण परेशानी

5.1 (TC: ऑप्शन मत पढ़िएÉ पेशंट को जवाब देने दीजिए। अगर जवाब नीचे के ऑप्शंज़ से मैच करे तो टिक करिएÉ अन्यथा लिखिए. एक से ज्यादा ऑप्शन चुन सकते हैं) ....
>>> Oues: जब आपको पता चला कि यह बीमारी हुई हैÉ तब आपके दिमाग में सबसे बड़ी चिंता क्या थी?
*

- [ ] महीनों दवाईयाँ खाने की दिक्कत
- [ ] ठीक होने की चिंता
- [ ] परिवार वालों की सुरक्षा
- [ ] नौकरी के जाने की चिंता
- [ ] घर के काम-काज कि ज़िम्मेदारी
- [ ] इलाज का खर्चा
- [ ] कोई चिंता नहीं, दवाई लेंगे, ठीक हो जाएँगे
- [ ] Other:

5..2 मैं आपको ऑप्शन पढ़ के सुना रहा हूं। रही हूँ É इनमें से .2 चुनिये जो आपकी सबसे बड़ी चिंताएँ थी *

- [ ] इलाज का खर्चा
- [ ] नौकरी के जाने की चिंता
- [ ] कोई चिंता नहीं, दवाई लेंगे, ठीक हो जाएँगे
- [ ] घर के काम-काज कि ज़िम्मेदारी
- [ ] ठीक होने की चिंता

Once the patient has selected options which suit him/her——> IN this questions, TC will read out options and ask patient to select top two.

Here, exactly 2 options need to be selected.

SECTION 6 - डायग्नोसिस के पहले का समय

(TC पढ़ के सुनाइए) अब मैं आपसे आपके इलाज शुरू होने के पहले के समय के बारे में कुछ सवाल करूँगा। करूँगी । कृपया निस्संकोच होकर जवाब दें ।

6.1 बीमारी पकड़ में आने से पहले आपका क्या हाल था? में 2 अलग अलग उदाहरण दे रहा हूँ, आपके लिये क्या सही बैठता है, बताईये। यदि इसके अलावा कोई उत्तर है तो संक्षेप में, 1-2 लाइन में बताइए *

- ○ सब ठीक था और अचानक खांसी हुई ।दर्द हुआ ।कुछ और चीज़ के लिए डॉक्टर के पास गए और उन्होंने सीधा बता दिया की यह हुआ है।
- ○ बहुत दिन से चल रही थी यह दिक्कत। काफ़ी सारे डॉक्टर के पास जा चुके थे, काफ़ी सारी दवाइयाँ ले चुके थे, पैसा भी काफ़ी लगा फिर भी ठीक नहीं हुआ। उसके बाद पता चला की यह बीमारी है।
- ○ Other:

6.2 जब से दिक्कत शुरू हुई थी, तब से अब तक कितने डॉक्टर को दिखाया होगा? अभी वाले डॉक्टर के अलावा (नम्बर रेकर्ड करें) *

Your answer

6.3 जब पहली बार कुछ लक्षण दिखे थे, तब से ले कर अभी वाले डॉक्टर की दवाई शुरू करने तक कितना समय लगा होगा? *

- ○ < =2 हफ़्ता
- ○ 1 महीना
- ○ 1.5 महीने
- ○ 2 महीने
- ○ 2.5 महीने
- ○ 3 महीने
- ○ 3.5 महीने
- ○ 4 महीने या ज्यादा (>=4)

Important Question:

If patient answers “0” doctors in earlier question (6.1) and selects 2 months here, then TC needs to confirm that the patient did not go to any doctor for 2 months, inspite of having symptoms.

Here, we wish to confirm if patient has understood the question and is answering correctly.

6.4 बीमारी की पहचान के बाद क्या आपने किसी और डॉक्टर को दिखाया थाई शायद दूसरी राय लेने के लिए? *

- Yes
- No
- सोचा था पर नहीं गए

Important Question:

This is whether patient went to another doctor after the current diagnosis (with the present doctor)

SECTION 7 - साइड इफ़ेक्ट्स

(TC पढ़ के सुनाइए) अब हम इलाज के दौरान हुए दवाइयों से दिक्कत के बारे में सवाल करेंगे `

7.0 क्या आपको लगता है कि आपको दवाएँ कुछ ज्यादा ही देर तक लेनी पड़ रही है और होंगी? *

- पूरी तरह सहमत
- सहमत
- ना सहमत ना असहमत
- असहमत
- पूरी तरह असहमत

7.1 इलाज के दौरान कभी आपको इनमे से कोई दिक्कत महसूस हुई? मैं ऑप्शन (option) पढ़ के सुनातीI सुनाता हूँÉ जो जो सही हैÉ बताना हाँ या नहींÉ ये भी बताना की कितने लम्बे समय तक चले   आप एक से ज्यादा भी हाँ बोल सकते है ĩ *

	No	I हफ़्ता या कम	2- 3 हफ़्ता	4 हफ़्ता	1.5 महीने	2 महीने	2 महीने से ज्यादा
उलटी	○	○	○	○	○	○	○
घबराहट	○	○	○	○	○	○	○
लाल पेशाब	○	○	○	○	○	○	○
जोड़ो में दर्द	○	○	○	○	○	○	○
दस्त	○	○	○	○	○	○	○
खुजली	○	○	○	○	○	○	○
बहुत गर्मी लगना और पसीना आना	○	○	○	○	○	○	○
Ottherr.	○	○	○	○	○	○	○

In a smartphone, these options are not visible unless one scrolls to the right.

7..2 इनके निवारण के लिए आपने अक्सर क्या कियाi मैं ऑप्शन पढ़ के सुनाती/सुनाता हूँi जो जो सही हैi बताना हाँ या नहींååå यहाँ भी एक से ज्यादा आप बता सकते है

- [ ] कुछ नहीं
- [ ] घरेलू इलाज
- [ ] TC को फ़ोन किया
- [ ] डॉक्टर को फ़ोन किया
- [ ] तुरंत डॉक्टर को दिखाने गए
- [ ] Other:

7.3 TC: पेशेंट को बोलने दीजियेi और जो वो बोले रिकॉर्ड करे. खुद से ऑप्शंन/options ना दे . ... QUES: इसके अलावा आपके शरीर में दवाइयों के वजह से क्या कोई और दिक्कत हुई? *

Your answer

7.4 अब में कुछ और साइड इफेक्ट्स बोलूंगी।बोलूंगा। अगर आपको दवाई के कारण इनमें से कुछ भी महसूस हुआÉतो बताना हाँ या नहीं और कितने समय तक रहा। *

	No	<=2 हफ़्ता	1 महीना	1.5 महीने	2 महीने	2.5 महीने	3 महीने या ज्यादा
बाल झड़ना	○	○	○	○	○	○	○
त्वचा का काला पड़ना	○	○	○	○	○	○	○
दांतों में कल्पन	○	○	○	○	○	○	○
मुँहासे	○	○	○	○	○	○	○
बहुत थकान	○	○	○	○	○	○	○
और कोई	○	○	○	○	○	○	○

In a smartphone, these options are not visible unless one scrolls to the right.

7.[illegible] इनके निवारण के लिए आपने अक्सर क्या किया[illegible] में ऑप्शन पढ़ के सुनाती।सुनाता हूँ[illegible] जो जो सही है[illegible] बताना हाँ या नहीं--- यहाँ भी एक से ज्यादा आप बता सकते है। *

- ☐ कुछ नहीं
- ☐ घरेलू इलाज
- ☐ TC को फ़ोन किया
- ☐ डॉक्टर को फ़ोन किया या दिखाया
- ☐ दूसरे डॉक्टर को दिखाया
- ☐ अलग।देसी इलाज के तरीक़े ढूंढे
- ☐ Other:

Section 8: ---> अब में आपसे कुछ और घर के बारे में सवाल करूँगा करूँगी।

8.1 जो व्यक्ति घर चलाने में सबसे बड़ा योगदान देते हैं उन्होंने कहा तक पढ़ाई करी है ?

- पढ़ाई नहीं की (अनपढ़)
- 4 वीं कक्षा तक स्कूल
- 5 वीं कक्षा से 9 वीं कक्षा तक स्कूल
- 10 वीं कक्षा तक स्कूल
- 12 वीं कक्षा तक स्कूल
- डिप्लोमा
- अधूरा ग्राजुएशन
- ग्राजुएशन।कॉलेज
- पोस्ट ग्राजुएशन

THIS question is for the individual who is the primary financial contributor in patient's family. It is not necessarily the patient or the one answering the questions.

8.2 (TC - अगर पेशंट से बात हो तो उनकी पढ़ाई वरना स्पष्ट करें कि उत्तर पेशंट के लिए होना चाहिए) . ...Ques: आपने कहाँ तक पढ़ाई करी है?

- पढ़ाई नहीं की (अनपढ़)
- 4 वीं कक्षा तक स्कूल
- 5 वीं कक्षा से 9 वीं कक्षा तक स्कूल
- 10 वीं कक्षा तक स्कूल
- 12 वीं कक्षा तक स्कूल
- डिप्लोमा
- अधूरा ग्राजुएशन
- ग्राजुएशन।कॉलेज
- पोस्ट ग्राजुएशन

8.3 (TC - यदि पेशेंट की जगह किसी और से बात हो रही है तो उनके लिए पूछे) - आपने कहाँ तक पढ़ाई करी है?

- NA (पेशेंट से ही बात हो रही है)
- पढ़ाई नहीं की (अनपढ़)
- 4 वीं कक्षा तक स्कूल
- 5 वीं कक्षा से 9 वीं कक्षा तक स्कूल
- 10 वीं कक्षा तक स्कूल
- 12 वीं कक्षा तक स्कूल
- डिप्लोमा
- अधूरा ग्राजुएशन
- ग्राजुएशन।कॉलेज
- पोस्ट ग्राजुएशन

If someone else is answering questions on behalf of the patient (a family member); then this question asks for his/her education.

8..4 आपके।इनके घर में कितने कमरे हैं। रसोई को मत गिनियेगा (TC - कमरो कि संख्या रिकोर्ड करें) *

Your answer

[illegible] में कुछ वस्तुओ के नाम लूंगा/लूंगी, इनमें से बताना की आपके पास घर पर क्या हैं। अगर आपको अजीब लगे तो भी बता देना, general सवाल है सर्वे के लिए। *

	Yes	No
पंखा	○	○
रसोई गैस स्टोव ((LPG))	○	○
दो पहियों का वाहन जैसे की स्कूटर	○	○
रंगीन टी वी	○	○
फ्रिज ((ffrriidge))	○	○
वॉशिंग मशीन ((washiing machiine))	○	○
कंप्यूटर या लैपटॉप ((computterr/llapttop))	○	○
कार/जीप/वैन	○	○
एयर कंडीशनर ((AC))	○	○
Ellecttrriiciitty/बिजली	○	○

Click YES if the patient has the item on left in his/her house. Else, click NO.

TC - पेशंट से पूछे जाने सवाल ख़तम हो चुके है। आगे के प्रश्न आप अपने आप भरें जो पेशंट के साथ बिताए समय के हिसाब से हो

9.1 यदि इस पेशंट ने परिवार वालों को बिमारी के बारे में बताया है तो पेशंट के परिवार वालों का क्या नज़रिया रहा है ? *

- [ ] पेशंट को उचित खाना और आराम मिले इसका ध्यान रखते हैं
- [ ] पेशंट को काफ़ी जिम्मेदारियाँ ख़ुद ही देखनी पड़ती है
- [ ] पेशंट को परिवार में काफ़ी भेदभाव का सामना करना पड़ता है
- [ ] इसके बारे में पेशंट ने ज़्यादा नहीं बताया

From here-on, TC needs to answer questions. PATIENT questions are over.

.

9..2 जब इस पेशंट को साइड इफ़ेक्ट होते हैं तो आपके अनुसार पेशंट का कैसा व्यवहार रहता है *

- [ ] दिक्कत से छुटकारा पाने को तत्पर रहते हैं और अपने डॉक्टर को दिखाते हैं
- [ ] हर बार जब मुझसे बात होती है तो फिरसे इसके बारे में बोलते हैं
- [ ] इस् बात को जो समझते हैं की दिक्कत दवाइयों के वजह से हैं और इलाज खतम होने के बाद ठीक हो जाएगी
- [ ] पेशंट ने इस बारे में ज़्यादा बात नहीं की
- [ ] विशिष्ट दिक्कत से छुटकारा पाने के लिए अलग डॉक्टर को दिखाते हैं

9.3 क्या आपको इस पेशंट को लम्बे इलाज के बारे में समझाने में दिक्कत आयी *

- ○ yes
- ○ no
- ○ थोड़ी बहुत

9.4 लम्बे इलाज के बारे में समझाने पर इस पेशंट के साथ आपका अनुभव कैसा रहा *

- शुरुआत में समझाया था और समझ गया और दवाई ले रहा। रही है
- पेशंट को कई बार समझाना पड़ा था की कोर्स खतम करना ज़रूरी है। पर अभी दवाई लेती। लेता है
- हाँ, काफ़ी बार समझाया था, शुरू में भी, पर अभी दवाई कम ही लेती। लेता है
- पेशंट के बर्ताव से पता नहीं चल पा रहा है

9.5 क्या यह पेशंट बीमारी की गम्भीरता को समझते है और ख़ुद ही इलाज के बारे में सावधानी रखते है *

- yes
- no
- थोड़ा बहुत
- पेशंट के बर्ताव से पता नहीं चल पा रहा है
