# Supplementary material for: Adverse drug reactions in tuberculosis treatment: Incidence, duration and resolution pathways from a mixed-methods patient-centric study in India
Source: PLOS Glob Public Health. 2025 Dec 29;5(12):e0004149. doi: 10.1371/journal.pgph.0004149 (PMC12747411; doi:10.1371/journal.pgph.0004149)
Supplement: S4 Appendix — (PDF) [file pgph.0004149.s004.pdf]

A GUIDE TO

ADVERSE DRUG REACTION MANAGEMENT OF

TB PATIENTS

Joint Effort for Elimination of Tuberculosis

CONTENTS

CHAPTER I.

What is an Adverse Drug Reaction (ADR) 1

CHAPTER II.

Common ADRs and their management 3

Communicating with TB patients on ADR 10

CHAPTER III.

* Severity of ADRs
* Treatment Coordinators' role in ADR management
* Referring TB patients to doctors for ADR management 11

CHAPTER

1 WHAT IS AN ADVERSE DRUG REACTION

INTRODUCTION

Treatment of TB requires the use of multiple medications and most patients will experience some difficulty in tolerating them. The responses of individuals to these medications will vary, and therefore cannot be predicted. This manual will help you be better equipped to help handle the questions your patients may have on side effects and their management.

WHAT IS AN ADVERSE DRUG REACTION (ADR)?

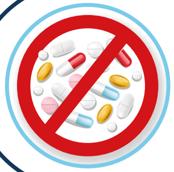

ADR is an unwanted or harmful reaction experienced after having a medicine and suspected to be caused due to the medicine.

Severity of adverse effect varies from tolerable and mild ADRs to serious and life threatening ADRs.

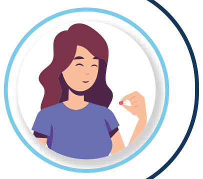

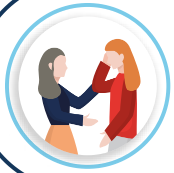

Every TB patient on anti TB treatment should be monitored closely for adverse drug reaction so that he/she does not stop the treatment.

Early detection and adequate management of ADRs is necessary for a good TB treatment outcome.

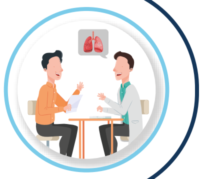

WHICH PATIENTS ARE MORE PRONE TO DEVELOP ADRs?

ADRs can happen to any TB patient. However, some patients might be more prone to develop ADRs.

- Those who consume alcohol while on anti TB treatment.
- Those who are simultaneously taking other medicines (homeopathic, ayurvedic, unani, etc.) for TB.
- Those who are suffering from other diseases such as diabetes, increased BP (hypertension), thyroid disorder, liver & renal diseases.
- TB patients living with HIV infection.
- Elderly TB patients.
- Those who are anemic and malnourished.
- Those who are not taking their medicines regularly.

CHAPTER

2 COMMON ADRs AND THEIR MANAGEMENT

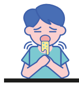

NAUSEA AND VOMITING

Stomach discomfort and the sensation of wanting to vomit.

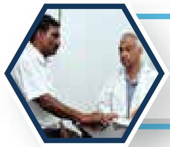

Counsel and reassure patients as it usually resolves with time.

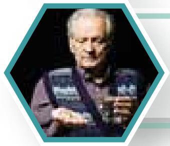

Suggest taking medicines after a gap of few minutes.

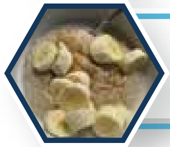

Advise patients to take drugs embedded in banana.

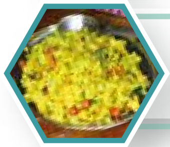

Advise patients to take a light meal before taking medicine.

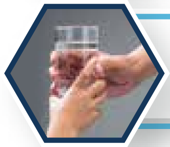

Ensure adequate hydration. Suggest usage of ORS if required.

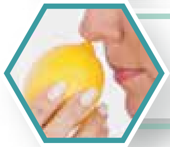

Suggest home remedies like smelling a lemon to ease discomfort. Antacid Aciloc or Rantac can be used which are available OTC after consultation with doctor.

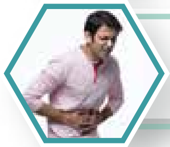

Refer to their treating doctor if nausea persists, blood in vomit and/or severe dehydration.

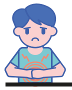

GASTRITIS AND PAIN IN ABDOMEN

Redness and swelling of the stomach lining leading to pain and discomfort.

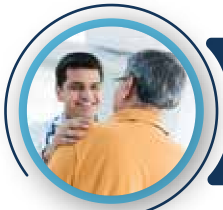

Counsel and reassure patients as it usually resolves with time.

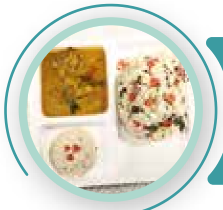

Suggest taking a light meal before taking medicine.

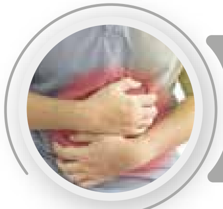

Refer to a doctor if there is severe abdominal pain or blood in vomit.

DIARRHEA

Loose, watery stool or a frequent need to have a bowel movement

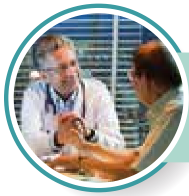

Counsel and reassure patients as it usually resolves with time.

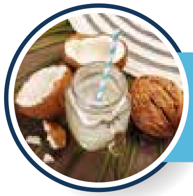

Encourage fluid intake (water, buttermilk, lemon water, and coconut water).

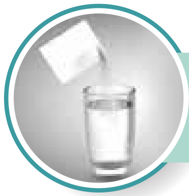

Give Oral Rehydration Solution and ask patient/ family to watch out for signs of dehydration.

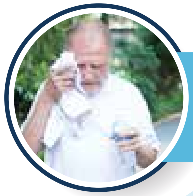

Refer to a doctor if patient develops dehydration.

HEADACHE AND GIDDINESS

Pain in the head or tendency to fall or stagger.

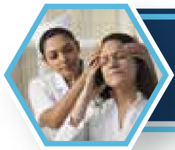

Counsel and reassure patients as it usually resolves with time.

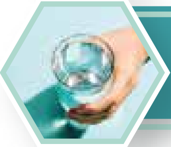

Encourage fluid intake.

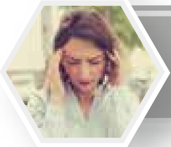

Refer to a doctor if the problem persists and/or severity increases.

SKIN RASHES, ITCHINESS AND ALLERGIC REACTIONS

Redness of skin leading to discomfort

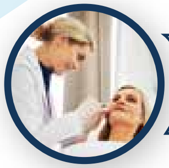

Counsel and reassure patients as reactions subside with time.

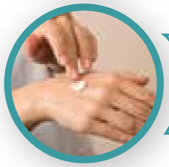

Suggest use of moisturizing cream for dry skin.

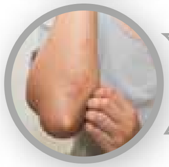

Refer to a doctor if there are serious allergic reactions/no improvement in symptoms or if the symptoms worsen.

PAIN IN JOINTS

Pain in knees, wrists, elbows and other joints

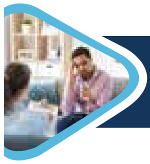

Counsel and reassure patients as it usually resolves with time.

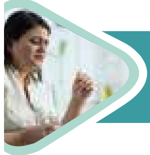

Counsel patient against taking Ibuprofen containing drugs. Instead Paracetamol (Crocin) can be taken to relieve discomfort.

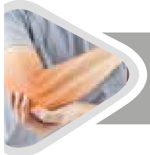

Refer to a doctor if more than 2 joints are involved, or pain is not relieved.

TINGLING, BURNING, NUMBNESS IN HANDS AND FEET

Unusual sensations in hands and feet like decrease in ability to feel or sense pressure or 'pins and needles' sensation.

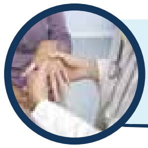

Counsel and reassure patients as it usually resolves with time.

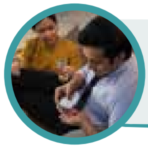

Ensure that patient is taking Pyridoxine. If the patient is not taking Pyridoxine and tingling and numbness continues, refer to treating physician.

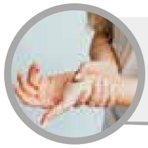

Refer to a doctor if the problem persists.

JAUNDICE

Yellowish discoloration of eyes, skin, mucosal surface

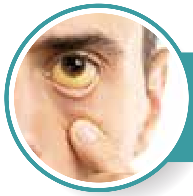

A relatively uncommon ADR.

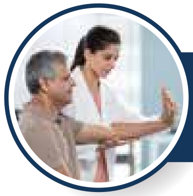

Patient should STOP all medications and consult the treating physician immediately.

FATIGUE AND TIREDNESS

Reduced energy, physical and mental exhaustion.

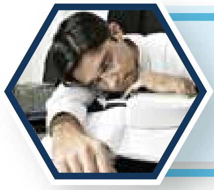

If the patient complains of fatigue and tiredness, especially in continuous phase (CP), advise patient to start doing light exercises, take short walks and sit in morning sunlight.

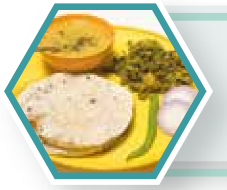

Emphasize on eating healthy and nutritious food.

HAIR FALL AND SKIN DARKENING

Excessive hair loss and changes in skin colour.

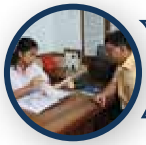

Acknowledge the worries of patient. Convey that you understand that it is difficult to accept the changes in body but patients should focus on fighting the disease.

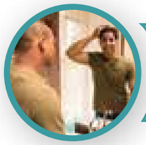

Reassure that on treatment completion, the body will repair itself and soon patients will start feeling better.

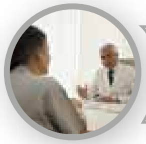

Patients can also seek advise from a specialist after completion of their treatment.

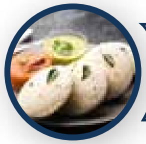

Emphasize on eating healthy and nutritious food.

COMMUNICATING WITH TB PATIENTS ON ADR

HELPFUL THINGS TO SAY / DO

- You look well / good today. How are you feeling?
- Mirror back what is being said. If the person says, "my back is really hurting me" Tell them, "Your back hurts, that must be difficult for you?
- Listen!
- Help patients understand what is going on
- Check if the patient met the doctor and how they are feeling
- Understand what the doctor has advised them to do
- Don't jump to conclusions
- Don't say it is no big deal; understand it's a big deal for the patient and their family
- Acknowledge their pain and / or difficulties but also, reassure that they are going to feel better soon
- Remind them that their struggle is temporary and is bound to end soon
- Reassure that patient is showing signs of improvement
- If unsure at any stage, please refer patients to their treating physician
- Involve family

SEVERITY OF ADRs, THEIR MANAGEMENT AND WHEN TO REFER TO A DOCTOR

Common ADRs	Treatment coordinator		Refer to Treating physician	
	Mild	Moderate	Severe	Potentially Severe
Nausea and vomiting	Nausea and vomiting with no symptoms of dehydration.	Nausea and vomiting with symptoms of dehydration like thirst, dizziness, tiredness, dry mouth and eyes, peeing little and less than 4 times a day.	Extreme vomiting, signs and symptoms of severe dehydration, electrolyte imbalance.	All signs and symptoms of severe and blood in vomit, altered level of consciousness.
Gastritis & pain in abdomen	Occasional discomfort, sour taste in mouth with acid reflux.	Burning sensation in upper abdominal region and at times acidity.	Severe pain in the abdomen, Acidity, burping, flatulence, vomiting.	All signs and symptoms of severe and blood in vomit, altered level of consciousness, electrolyte imbalance.
Diarrhea	2-3 loose stools, no signs and symptoms of dehydration.	3-10 loose liquid or watery stools with signs & symptoms of dehydration.	More than 10 watery stools, signs and symptoms of dehydration, electrolyte imbalance.	All signs and symptoms of severe; blood in stool, fever, intense abdominal pain, altered level of consciousness.
Tingling, burning, numbness in hands and feet	Mild numbness and weakness in hands and feet.	Prickling, stabbing, burning or tingling along with gradual increase in severity of numbness & weakness.	Signs and symptoms of moderate neuropathy, extreme sensitivity to touch.	Lack of coordination and balance, muscle weakness, poor control on bowel & bladder.
Pain in joints	Pain on touching joint.	Pain on walking and swelling & redness, warmth in & around joint.	Stiffness, signs of increased tenderness.	All signs and symptoms of severe; weakness, restricted joint movement.
Skin rashes, itchiness, and allergic reactions	Itching, skin rashes.	Itching and skin rashes with tingling and burning sensations.	Itching, with increased size and raised wheels swelling of lips & tongue.	Severe and potentially life-threatening allergic reaction or a serious disorder of the skin with painful rashes or shedding of skin.

Joint
Effort for
Elimination of
Tuberculosis
