# Supplementary material for: Adverse drug reactions in tuberculosis treatment: Incidence, duration and resolution pathways from a mixed-methods patient-centric study in India
Source: PLOS Glob Public Health. 2025 Dec 29;5(12):e0004149. doi: 10.1371/journal.pgph.0004149 (PMC12747411; doi:10.1371/journal.pgph.0004149)
Supplement: S5 Appendix — (PDF) [file pgph.0004149.s005.pdf]

टीबी की दवाइयों से जुड़े विपरीत एवं दुष्प्रभाव और उनके प्रबंधन एवं निवारण के लिए मार्गदर्शन

Joint Effort for Elimination of Tuberculosis

विषय-वस्तु

अध्याय 1.

दवा के विपरीत एवं दुष्प्रभाव (ADR) किसे कहते हैं? 1

अध्याय 2.

दवा के मामूली / सामान्य विपरीत एवं दुष्प्रभाव और उनके कुछ उपाय 3

दवा के विपरीत और दुष्प्रभाव के बारे में टीबी के मरीज़ों से बातचीत कैसे की जाए ? 10

अध्याय 3.

* दवा के विपरीत और दुष्प्रभावों (ADR) की गंभीरता
* ADR प्रबंधन में ट्रीटमेंट कोऑर्डिनेटर / ट्रीटमेंट साथी की भूमिका
* ADR प्रबंधन के लिए टीबी के मरीज़ों को डॉक्टर के पास रेफेर करने से जुड़ी जानकारी

11

अध्याय

1 दवा के विपरीत और दुष्प्रभाव (Adverse Drug Reaction) क्या होते हैं ।

परिचय

टीबी के इलाज में कई प्रकार की दवाओं का इस्तेमाल करने की जरुरत पड़ती है और अधिकतर मरीज़ों को इन्हें बरदाश्त करने में कुछ कठिनाई हो सकती है। हमें यह याद रखना है कि हर मरीज़ अलग है और इसलिए इन दवाओं का विपरीत असर भी अलग-अलग ही होगा। हमारे लिए पहले से कुछ भी पता लगाना मुश्किल है। यह गाइड आपको आपके मरीज़ों की दवा के विपरीत एवं दुष्प्रभावों से संबंधित सवालों के जवाब और उनके उपाय ढूँढने में मदद करेगी ताकि आप दवा के वपिरीत और दुष्प्रभावों और उनके प्रबंधन एवं निवारण के बारे में पूछे जाने वाले प्रश्नों का जवाब दे सकें।

दवा के विपरीत और दुष्प्रभाव (ADR) क्या होते हैं ?

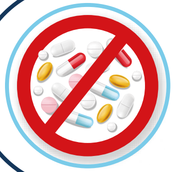

दवा का विपरीत और दुष्प्रभाव **(ADR)** किसी दवा खाने के बाद शरीर में होने वाली अवांछित **(unwanted)** या नुकसानदायक प्रतिक्रिया को कहते हैं जो संभवत: दवा के कारण होती है।

हालांकि दवा के कुछ विपरीत और दुष्प्रभाव आसानी से ठीक हो जाते हैं **(mild ADRs)**, जबकि कुछ के सही इलाज के लिए समय से डॉक्टर के पास जाना अनिवार्य होता है। हम ऐसे प्रभावों को **'life threatening'** बोल सकते हैं।

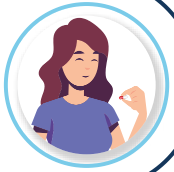

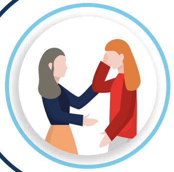

आपको हर टीबी मरीज़ के इलाज के दौरान इस बात का ध्यान रखना जरुरी है कि वह इन विपरीत और दुष्प्रभावों के कारण अपना ट्रीटमेंट बीच में न रोक दें।

ADRs की शुरुआत में ही पहचान करना और उसका पर्याप्त प्रबंधन करना टीबी के उपचार के अच्छे परिणाम के लिए जरुरी है।

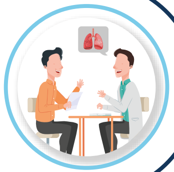

किस प्रकार के मरीज़ को ADRs होने का अधिक खतरा होता है?

ADRs किसी भी टीबी के मरीज़ को हो सकते हैं। हालांकि कुछ मरीज़ों को ADRs होने का अधिक खतरा हो सकता है।

वे मरीज़ जो टीबी के इलाज के दौरान शराब पीते है।

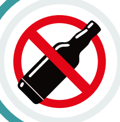

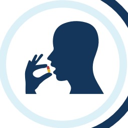

वे मरीज़ जो टीबी के लिए अन्य दवाएँ (होमियोपैथक, आयुर्वेदिक, यूनानी आदि) भी साथ में लेते है।

वे मरीज़ जिन्हें अन्य बीमारी जैसे डायबिटीज, उच्च रक्तचाप (High Blood Pressure), थायराइड, लीवर और गुर्दे का रोग भी है।

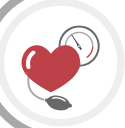

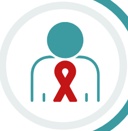

एचआईवी (HIV) संक्रमण से पीड़ित टीबी का मरीज़।

टीबी का बुजुर्ग मरीज़।

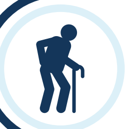

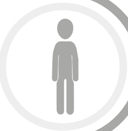

वे मरीज़ जो एनीमिया से ग्रस्त और कुपोषित है।

वे मरीज़ जो अपनी दवाएँ नियमित रुप से नही खा रहे है।

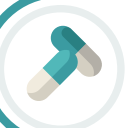

अध्याय

2 सामान्य ADRs और उनका प्रबंधन

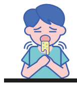

जी मिचलाना और उल्टी

पेट में तकलीफ और उल्टी महसूस होना।

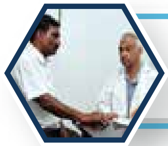

मरीज़ को सलाह और आश्वासन दें कि यह कुछ समय के बाद ठीक हो जाता है।

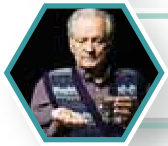

कुछ मिनट के अंतराल पर दवाएँ लेने की सलाह दें।

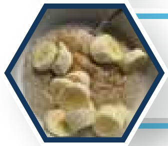

मरीज़ को दवा को केले में रखकर खाने की सलाह दें।

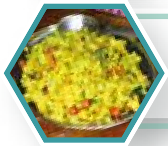

मरीज़ को दवा लेने से पहले हल्का भोजन करने की सलाह दें।

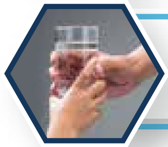

शरीर में पानी की पर्याप्त मात्रा बनाए रखना जरुरी है। जरुरत पड़ने पर ओआरएस (ORS) का घोल पीने की सलाह दें।

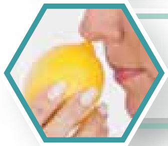

घरेलू नुस्खे जैसे-तकलीफ कम करने के लिए नीबू को सूँघने के लिए बताएँ। डॉक्टर की सलाह लेकर एसिलोक (Aciloc) और रैनटैक (Rantec) जैसे एंटासिड भी लिए जा सकते है।

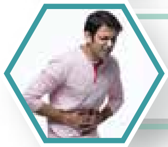

यदि उल्टी की तकलीफ बनी रहती है, उल्टी में खून आता है और / या गंभीर तौर पर शरीर में पानी की कमी होती है तो इलाज करने वाले डॉक्टर के पास भेजे।

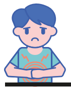

गैस्ट्राइटिस और पेटदर्द

पेट में सूजन होने से दर्द और तकलीफ होती है।

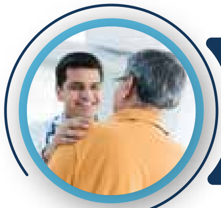

मरीज़ को सलाह दें और आश्वासन दें कि अक्सर यह धीरे-धीरे ठीक हो जाता है।

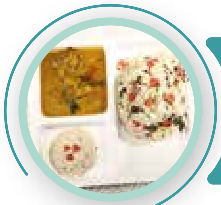

दवा लेने से पहले हल्का भोजन करने का सुझाव दें।

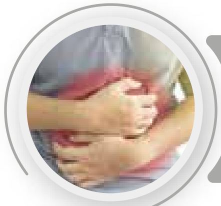

यदि पेटदर्द बहुत ज्यादा हो रहा है या उल्टी में खून आ रहा है तो डॉक्टर के पास भेजे।

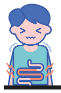

डायरिया

पतला, पानी जैसा मल होना या बार-बार शौच जाना

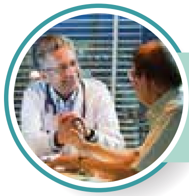

मरीज़ को सलाह दें और उसे आश्वासन दें कि धीरे-धीरे यह ठीक हो जाता है।

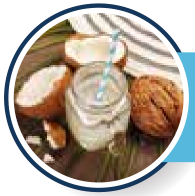

तरल पदार्थ (जल, छाछ, नीबू पानी, और नारियल पानी) ज्यादा से ज्यादा पीने के लिए कहें।

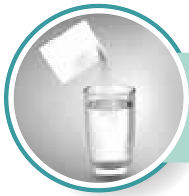

ओरल रिहाइड्रेशन सोल्यूशन (ओआरएस) दें और मरीज़ / परिवार से डिहाइड्रेशन के लक्षण पर ध्यान देने के लिए कहें।

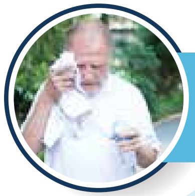

यदि मरीज़ को डिहाइड्रेशन होता है तो उसे डॉक्टर के पास भेजें।

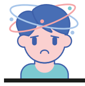

सिरदर्द होना और चक्कर आना

सिरदर्द होना या गिरने की आशंका होना या लड़खड़ाना

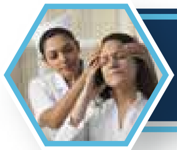

मरीज़ को सलाह दें और उसे आश्वासन दें कि अक्सर यह धीरे-धीरे ठीक हो जाता है।

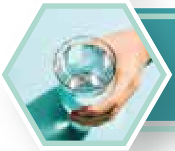

तरल पदार्थ ज्यादा लेने के लिए कहें।

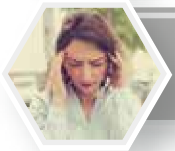

यदि समस्या बनी रहती है और/या गंभीरता बढ़ने लगती है तो मरीज़ को डॉक्टर के पास भेजें।

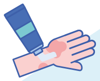

त्वचा पर लाल चक्ते पड़ना, खुजली और एलर्जी होना

त्वचा लाल होने से तकलीफ बढ़ जाती है।

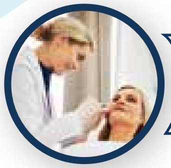

मरीज़ को सलाह दें और उसे आश्वस्त करें कि अक्सर धीरे-धीरे रिएक्शन ठीक हो जाता है।

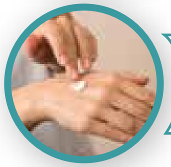

सूखी त्वचा के लिए माइश्चराइजिग (moisturizing) क्रीम का इस्तेमाल करने के लिए कहें।

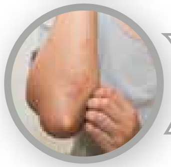

यदि गंभीर एलर्जिक रिएक्शन होता है/कोई सुधार नही होता है या स्थिति ज्यादा खराब हो जाती है तो डॉक्टर के पास भेजें।

जोड़ों का दर्द

घुटनों, कलाई, कोहनी और अन्य जोड़ों में दर्द

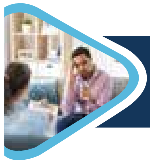

मरीज़ को सलाह दें और उसे समझाएँ कि अक्सर यह धीरे-धीरे ठीक हो जाता है।

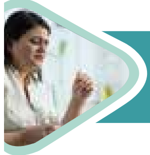

मरीज़ को आइबुप्रोफेन **(Ibuprofen)** वाली दवाएँ न लेने की सलाह दें। इसके बजाए वे तकलीफ दूर करने के लिए पैरासिटामॉल (क्रोसिन) ले सकते है।

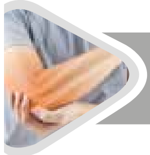

यदि दो से अधिक जोड़ों में दर्द होता है या दर्द ठीक नही हो रहा है तो मरीज़ को डॉक्टर के पास भेजें।

हाथों और पैरों में झुनझुनाहट, जलन, और उनका सुन्न हो जाना

हाथों और पैरों में झुनझुनाहट होना, कुछ भी महसूस न होना बल्कि सूई चुभने का एहसास होना।

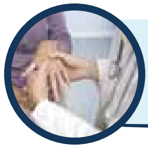

मरीज़ को सलाह दें और उसे आश्वस्त करें कि अक्सर यह धीरे-धीरे ठीक हो जाता है।

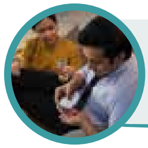

यह सुनिश्चित करें कि मरीज़ पाइरिडॉक्सिन **(Pyridoxine)** ले रहा है या नही। यदि वह पाइरिडॉक्सिन नही ले रहा है और उसे झुनझुनाहट और सुन्नपन की समस्या हो रही है तो उसे इसका उपचार करने वाले डॉक्टर के पास भेजें।

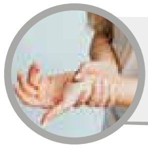

यदि यह समस्या बनी रहती है तो उसे डॉक्टर के पास भेजें।

पीलिया

आँखों, त्वचा, श्लैष्मिक सतह **(mucosal)** का पीला पड़ना

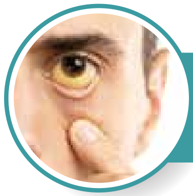

यह ADR बहुत ही कम मरीज़ में देखा जाता है।

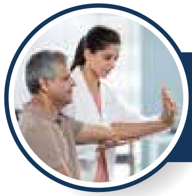

रोगी को सभी दवाएँ लेनी बंद कर देनी चाहिए और तुरंत उपचार करने वाले डॉक्टर से संपर्क करने की सलाह दें।

सुस्ती और थकान

शरीर की ऊर्जा कम होना, शारीरिक और मानसिक थकान

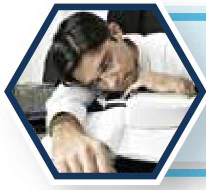

यदि मरीज़ विशेष रुप से लगातार सुस्ती और थकान की शिकायत करता है, तो रोगी को हल्का व्यायाम करने, थोड़ा टहलने और सुबह की धूप लेना शुरु करने के लिए कहें।

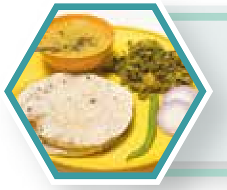

स्वस्थ और पौष्टिक खाद्य पदार्थ खाने पर जोर दें।

बालों का झड़ना और त्वचा का रंग काला पड़ना

बहुत अधिक बाल झड़ना और त्वचा का रंग बदलना।

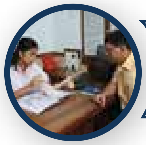

मरीज़ की चिता पर ध्यान दें। उसे बताएँ कि आप समझते है कि शरीर में होने वाले बदलाव को स्वीकार करना मुश्किल है लेकिन रोगी को इस रोग से लड़ने पर ध्यान देना चाहिए।

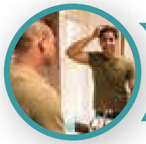

उसे आश्‍वस्त करें कि उपचार पूरा होने के बाद, शरीर खुद अपने आप पहले जैसा हो जाएगा और जल्द ही मरीज़ बेहतर महसूस करने लगेगा।

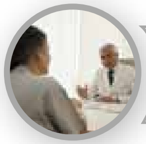

मरीज़ उपचार पूरा होने के बाद किसी विशेषज्ञ से भी सलाह ले सकता है।

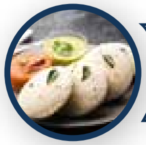

स्वस्थ और पौष्टिक भोजन करें।

दवा के विपरीत और दुष्प्रभाव के बारे में टीबी के मरीज से बातचीत कैसे की जाए?

कहने / करने के लिए उपयोगी चीजें

- आज आप पहले से बेहतर लग रहे है। आपको कैसा महसूस हो रहा है / लग रहा है?
- मरीज़ की तकलीफ को समझने की कोशिश करें, यह ना कहे कि यह छोटी से चीज है, बल्कि उन्हें कहे कि आप समझ सकते है कि वो कितनी परेशानी से गुजर रहे है।
- मरीज़ की बात को ध्यान से सुनें!
- जो हो रहा है उसे समझने में मरीज़ों की मदद करें।
- चेक करें कि क्या मरीज़ डॉक्टर के पास गया था और वह कैसा महसूस कर रहा है।
- यह समझे कि डॉक्टर ने उन्हे क्या करने की सलाह दी है।
- तुरंत निष्कर्ष न निकाले।
- यह न कहे कि यह कोई बड़ी बात नही है; बल्कि यह समझे कि यह मरीज़ और उसके परिवार के लिए बड़ी बात है।
- उनके दर्द और / या परेशानियों को समझे लेकिन यह भी आश्‍वासन दें कि वे जल्द ही बेहतर महसूस करेंगे।
- उन्हे याद दिलाएँ कि उनका यह संघर्ष अस्थायी है और यह जल्द ही खत्म हो जाएगा।
- मरीज़ को आश्‍वस्त करें कि उसके अंदर सुधार के लक्षण दिख रहे है।
- यदि आप किसी चरण पर अनिश्चित है, तो कृपया मरीज़ को उसके उपचार करने वाले फिजीशियन के पास भेजें।
- मरीज़ के परिवार को शामिल करें।

अध्याय

3 ADRs की गंभीरता, उसका प्रबंधन और रोगी को डॉक्टर के पास कब भेजें

सामान्य ADRs	उपचार समन्वयक		उपचार करने वाले डॉक्टर के पास भेजे	
	हल्का	मध्यम	गंभीर	संभावित रुप से गंभीर
जी मिचलाना और उल्टी	जी मितली और उल्टी लेकिन डिहाइड्रेशन का कोई लक्षण नही	जी मितली और उल्टी डिहाइड्रेशन के लक्षण जैसे प्यास लगना, चक्कर आना, थकावट, मुँह और आँख सूखना, पेशाब थोड़ी होना तथा दिन में 4 बार से कम होना	बहुत ज्यादा उल्टी होना, गंभीर डिहाइड्रेशन के लक्षण होना, इलेक्ट्रोलाइट का असंतुलित होना	गंभीरता के सभी लक्षण होना तथा उल्टी में खून आना और चैतन्यता (consciousness) का स्तर बदल जाना
गैस्ट्राइटिस और पेटदर्द	कभी-कभी तकलीफ होना, मुँह का खट्टा स्वाद होना तथा एसिड आना	पेट के ऊपरी हिस्से में जलन होना तथा एसिडिटी होना	पेट में गंभीर दर्द, एसिडिटी, डकार आना, पेट फूलना, उल्टी होना	गंभीरता के सभी लक्षण होना, और उल्टी में खून आना, चैतन्यता (consciousness) का बदला स्तर, इलेक्ट्रोलाइट का असंतुलित होना
डायरिया	2-3 बार पतला मल/ पखाना/ स्टूल होना, डिहाइड्रेशन का कोई लक्षण न दिखना	3-10 बार पतला मल/ पखाना/ स्टूल होना तथा डिहाइड्रेशन के लक्षण दिखना	10 से अधिक बार पतला मल/ पखाना/ स्टूल होना, डिहाइड्रेशन के लक्षण दिखना, इलेक्ट्रोलाइट का असंतुलित होना	गंभीरता के सभी लक्षण दिखना; मल में खून आना, बुखार, बहुत तेज पेट दर्द, चैतन्यता (consciousness) का बदला स्तर
हाथो और पैरो मे झुनझुनी, जलन, सुन्नपन होना	हाथो और पैरो में हल्का सुन्नपन और कमजोरी होना	चुभन, भयानक दर्द, जलन या झुनझुनी तथा सुन्नपन और कमजोरी धीरे-धीरे बढ़ना	मध्यम तंत्रिका (moderate neuropathy) विकृति के लक्षण होना, छूने के प्रति बहुत अधिक संवेदनशील होना	समन्वय और संतुलन में कमी, मांसपेशियो का कमजोर होना, पेट और मूत्राशय पर नियंत्रण कम होना
जोड़ो में दर्द	जोड़ो को छूने पर दर्द	चलने में दर्द और सूजन तथा लाली, जोड़ो में और उसके आसपास गर्म महसूस होना	अकड़न, जोड़ो का बहुत ज्यादा कोमल हो	गंभीरता के सभी लक्षण; कमजोरी, जोड़ो का ठीक से काम न करना
त्वचा पर लाल चकत्ते पड़ना, खुजली होना और एलर्जिक रिएक्शन होना	खुजली होना, त्वचा पर लाल चक्ते पड़ना	खुजली होना और लाल चक्ते पड़ना तथा झुनझुनी होना और जलन होना	खुजली, चक्ते का आकार बढ़ना, और जीभ की सूजन	गंभीर एलर्जिक रिएक्शन (allergic reaction) होना, त्वचा का झड़ना और चक्ते पड़ना, साथ ही गंभीर दर्द होना।

Joint
Effort for
Elimination of
Tuberculosis
